# Informing indicator species selection by combining species associations and environmental responses

**DOI:** 10.1002/eap.70296

**Published:** 2026-08-20

**Authors:** Tuuli Rissanen, Jukka Sirén, Raisa Mäkipää, Jarno Vanhatalo, Anna‐Liisa Laine

**Affiliations:** ^1^ Research Centre for Ecological Change, Department of Organismal and Evolutionary Biology, Faculty of Biological and Environmental Sciences University of Helsinki Helsinki Finland; ^2^ Institute of Biotechnology, Helsinki Institute of Life Science University of Helsinki Helsinki Finland; ^3^ Natural Resources Institute Finland Helsinki Finland; ^4^ Department of Mathematics and Statistics, Faculty of Science University of Helsinki Helsinki Finland

**Keywords:** biodiversity, boreal forest, climate, ecological monitoring, indicator species, vascular plants

## Abstract

Biodiversity is changing at an unprecedented pace, underscoring the need for effective and scalable ecological monitoring. Yet selecting which species to monitor remains a major challenge. Indicator species can offer cost‐effective proxies for broader biodiversity trends, but there is a critical gap in robust, data‐driven approaches to identify them across complex and rapidly changing ecosystems shaped by climate, land management, and their interactions. We provide a model‐based approach to select potential indicator species to monitor boreal understory communities, which are crucial part of forest biodiversity but often not as continuously monitored as trees. We focus on vascular plants due to their associations with multiple taxa. With joint species distribution models, we investigate species–species and species–environment relationships across Finland, utilizing nationwide understory vegetation data covering nearly 3000 study sites across different soil types and bioclimatic zones together with key environmental factors representing climate, habitat, and forest management. Species' associations and environmental responses differed between mineral soils and peatlands and across bioclimatic zones. We ranked species based on their association with other species and environmental responsiveness and observed that some species responded strongly to both, whereas many species were more related to one aspect than the other. Furthermore, we show that considering the effect of forest conditions together with the climate variables is important as they represent interdependent effects on biodiversity. Our study provides a robust framework for selecting indicator species that could be used in a wide range of biodiversity monitoring purposes and ecosystems, such as national forest monitoring programs. Combining information on species co‐occurrences and environmental responses allows identifying species that are informative of both local community composition and broader environmental change supporting more informed and efficient indicator selection.

## INTRODUCTION

Biodiversity is undergoing unprecedented change globally driven by climate change, land management practices, overexploitation, pollution, and invasion of alien species. Our ability to detect and predict biodiversity change depends on reliable information on the status and changes in species, communities, and ecosystems (IPBES, 2019). The need for long‐term ecological monitoring programs to track and understand dynamics in species populations and communities, assess species' conservation status, and inform management decisions is well acknowledged (Lindenmayer & Likens, 2010; White, 2019). However, biodiversity monitoring is heavily biased both geographically and across taxa as many specious taxonomic groups, such as plants and invertebrates are poorly covered globally (Moussy et al., 2021). Monitoring entire communities is inherently challenging due to the sheer number of species, variation in detectability, and resource constraints (Lindenmayer & Likens, 2010; Sato et al., 2019). One solution to overcome these challenges and to increase the temporal frequency of monitoring is to use surrogates—species or groups that act as proxies for broader biodiversity patterns—which can simplify monitoring by reducing the effort and resources needed (Bal et al., 2018; Sato et al., 2019). Such indicator species are expected to predict key ecological characteristics such as community composition, habitat type, or environmental conditions based on the species' niche preferences.

Indicator species, whether single species or species groups, can be selected using a variety of approaches, each with its own strengths and limitations. Common methods include expert‐based selection, where species are chosen based on ecological knowledge or conservation relevance (McGeoch et al., 2002); statistical associations with environmental variables, which identify species strongly linked to specific conditions (De Cáceres et al., 2010); and multivariate techniques that assess species fidelity and specificity to habitats or community types (Dufrêne & Legendre, 1997; Ricotta et al., 2021). More recently, machine learning and species distribution models have been used to identify indicators across large datasets (Bal et al., 2018; Khan et al., 2016). However, many approaches overlook species co‐occurrence patterns, potentially missing key community‐level interactions, even though biodiversity is simultaneously shaped by both abiotic and biotic processes. Biotic interactions, both positive and negative, play a significant role in shaping species distributions even after accounting for abiotic filtering (Blanchet et al., 2020; Wisz et al., 2013). This emphasizes the usefulness of accounting for the associations among species when identifying potential indicators as the presence of both a strong facilitator and a competitor species could predict the composition of the surrounding community. Additionally, variations in species associations can reflect abiotic conditions as species niche preferences differ (Heikkinen & Mäkipää, 2010). Moreover, in terms of abiotic conditions, species distributions may simultaneously be shaped by different anthropogenic pressures (Speißer et al., 2022), such as climate and management impact, whose effects on biodiversity are interdependent and often nonadditive (Montràs‐Janer et al., 2024). Intensification of land use across Europe has led to widespread biodiversity loss and species homogenization (Ollerton et al., 2014), while concurrent climate warming has driven species' range shifts (Antão et al., 2022). Therefore, integrating both climate and land management variables is critical for identifying indicator species that reliably reflect broader biodiversity patterns under global change.

Boreal forests are exposed to environmental changes especially because of forest management and habitat fragmentation caused by forestry, agriculture, and other human practices (Curtis et al., 2018; Eggers et al., 2022), but they are also susceptible to climate change (Reich et al., 2022). Notably, natural disturbances, such as drought, windstorms, and insect and pathogen outbreaks, can be heavily affected by change in climatic conditions and land management (Venäläinen et al., 2020). In addition to tree‐level responses, alterations in silvicultural practices and forest structure influence forest understory species (Hedwall et al., 2013), which are often more sensitive to environmental changes than the overstory species (Gilliam, 2007). For example, the occurrence of bilberry (*Vaccinium myrtillus*), one of the boreal forest keystone species, is highly affected by the forest age and structure reflecting the disturbance history of the forest (Hedwall et al., 2013). Moreover, the understory hosts the majority of forest biodiversity, has a crucial role in ecosystem functions, such as productivity and biogeochemical cycles, and can affect the overstory seedling dynamics (Thompson et al., 2011). Therefore, to understand, estimate and monitor the status of a forest ecosystem, the understory should not be overlooked. Yet, forest monitoring focuses heavily on tree stands and tree‐level responses. For example, in Finland, extensive long‐term data on forest structure and management have been collected through the National Forest Inventory (NFI) since the 1920s (Korhonen et al., 2024), and in the current inventory design, roughly 9400 sample plots are surveyed annually. In contrast, nationwide understory inventories mapping the whole understory community from ~3000 sample plots have been carried out only three times since the 1980s (Tonteri et al., 2016, 2026).

Including understory species in continuous forest monitoring schemes would enhance detecting and mapping of changes in forest biodiversity in response to climate and management practices. Here, indicator species could offer a feasible solution to produce temporally more continuous biodiversity information to complement the surveys of the whole understory and the tree measurements of the NFI. Vascular plants could be especially useful indicators as they host a great proportion of forest biodiversity and are associated with multiple taxa such as mosses and lichens (Jokela et al., 2018), beetles (Schuldt & Assmann, 2010), and butterflies (Ekroos et al., 2013). Moreover, vascular plants are relatively easy to identify in the field even to species‐level, which is important for the applicability of indicator species for long‐term monitoring schemes such as NFI (Sato et al., 2019). In this study, focusing on Finland, we present a model‐based approach to identify potential indicator species for mapping boreal forest understory communities. Specifically, we aim to find species whose occurrences are both associated with those of other species reflecting biotic interactions in the community and responsive to key environmental conditions reflecting the effect of abiotic conditions and possible changes in them. To achieve this, we apply joint species distribution models (Norberg et al., 2019), which allow us to account for species–environment relationships and interspecific associations simultaneously offering a scalable and data‐driven framework for more informed and efficient indicator selection. Integrating the identified indicator species into nationwide forest monitoring would support more continuous and cost‐effective biodiversity monitoring in the future.

## MATERIALS AND METHODS

### Study area

**FIGURE 1:**
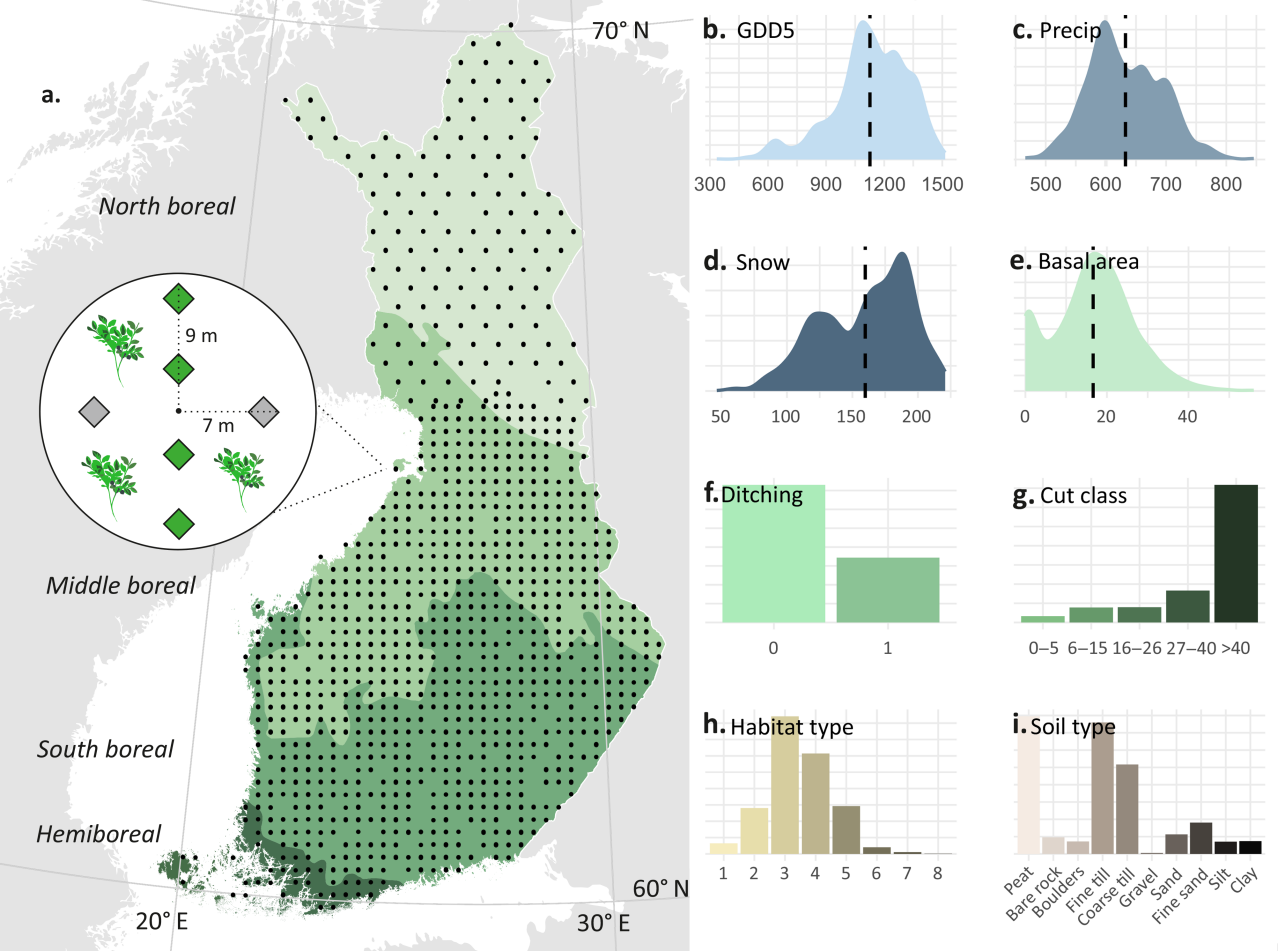
Study area and the environmental variables. Panel (a) shows study site locations (*n* = 2898) across different bioclimatic zones and vegetation sampling design on each site. Vegetation survey is done on the green quadrats and complemented with the gray ones if some of the green ones could not be used. Variation in environmental variables is visualized across mineral and peatland sites and all bioclimatic zones using data from 2685 sites from which all environmental variables were available. Density plots with dashed line showing the mean value visualize variation in continuous variables (b–e) and bar plots in categorical variables (f–i). GDD5 = growing degree days. Ditching: 0 = not ditched, 1 = ditched. Cut class: Time since clear cut in years. Habitat type varies from nutrient richest (1) to nutrient poorest (8) both on mineral and peatland sites (see Appendix S1: Table S3 for detailed information).

Our study spans Finland located ca. 60–70° N and 20–31° E, covering almost the entire country along a ~1200‐km latitudinal gradient (Figure 1a). Climate in Finland is strongly influenced by the Polar Front and the North Atlantic Current which drive temperature and precipitation patterns and their seasonality. Additionally, topographic variability, a large number of lakes and wetlands, as well as varying elevation (~0–1000 m above sea level [asl]) shape the environmental conditions (Tikkanen, 2005). Mean annual temperature ranges from −2.2 to 7.1°C and mean annual precipitation is around 609 mm (period 1991–2020), but precipitation patterns vary across different parts of the country (Jokinen et al., 2021). Finland can be divided into four bioclimatic zones ranging from hemiboreal southern coast to north boreal zone (Ahti et al., 1968), but in this study, the hemiboreal zone is combined with south boreal zone due to its restricted size. Forests cover approximately 75% of the land area and across Finland mesic heath forest is the most common forest habitat type characterized by Norway spruce (*Picea abies*) in the overstory and bilberry (*V. myrtillus*) in the understory. However, towards north, Scots pine (*Pinus sylvestris*) and lingonberry (*Vaccinium vitis‐idaea*) become more dominant. The Finnish forests grow both on mineral and peatland soils with the coverage of ~65% and ~35% of the forest area, respectively (Korhonen et al., 2024).

### Understory vegetation data

The vegetation data used in this study were taken from the most recent national understory monitoring that was carried out in 2021–2023 by the Natural Resources Institute Finland (Tonteri et al., 2026). The systematic sampling network consists of permanent study sites which were established in 1985 in connection with the 8th Finnish NFI (Tonteri et al., 2016). In the original data, there were 2898 sites of which 1882 sites were located on mineral soils and 1016 on peatlands. On each site understory vegetation is surveyed from four 2‐m^2^ quadrats along a south–north oriented transect within a circular 300‐m^2^ plot (Figure 1a). From each quadrat, the coverage of vascular plants (0.1%–100%) is visually estimated in 1% increments (cover value of 0.1 is given if a species is present but does not cover 1%), and the plants are identified to species level (except some ambiguous species on genus level) (Kaarlejärvi et al., 2021). In our analysis, we used species' presence–absence data on quadrat level to maximize the variation in species communities (7467 quadrats on mineral soils and 4024 on peatlands). Species pool comprised of 84 and 66 species in mineral and peatland sites, respectively. On mineral sites species number was highest in the south boreal zone with 71 species followed by middle and north boreal zones with 40 and 32 species. In peatland sites, middle boreal zone had more species than south and north boreal zones with 54, 45, and 40 species, respectively. To consider how easy the potential indicator species are to identify in the field, species were classified to three identifiability categories from easy to difficult based on expert knowledge (Appendix S1: Tables S1 and S2).

### Climate and forest data

To identify potential indicator species based on their environmental responsiveness we utilized both climate and forest variables. We used three climate variables to account for summer and winter conditions as well as variation in precipitation. Growing degree days, with a 5°C threshold, (GDD5, Figure 1b) was used to describe growing season temperature sum by summing daily mean temperatures that exceeded 5°C. Annual precipitation sum (in millimeters, Figure 1c) was calculated from monthly means and annual snow cover length (Figure 1d) was calculated from daily snow cover measurements as the number of days when snow cover depth was >0 cm. All climate variables were calculated as a mean of a five‐year period preceding the start of the vegetation survey (i.e., 2016–2020) and derived from a gridded climate dataset, provided by the Finnish Meteorological Institute (Aalto et al., 2016).

In addition to the climate factors, we used five variables to represent forest structure, management practices, and site habitat type and fertility. Basal area (in square meters per hectare, Figure 1e) describes how much of the site surface area is covered by tree stems, and it can be used as a proxy for forest age and stand density and as an estimate for understory light availability (Tonteri et al., 2016). Basal area is based on stem diameter measurements at 1.3 m height. Ditching (Figure 1f) describes the drainage status of the site, that is, whether the site is ditched or not, representing one of the main disturbances in Finnish forests affecting both the understory vegetation and trees. Cut class (Figure 1g) is another management related variable reporting the time since the last regeneration cut (i.e., clear‐cut). Management type (e.g., clear‐cut, thinning, or selection cut) has been reported on each site during the national understory surveys in 1985, 1995, and 2021–2023 for the past 10 years (26 years in the latest survey) which enabled detecting the management history for a 1975–2023 period. Using this combined history, we detected when the last clear‐cut, if any, had happened as it represents both a major disturbance and the age of the forest. Time since clear‐cut was categorized into five categories as time was not reported continuously in all surveys. Habitat type (Figure 1h) accounts for variation in different forest habitat types, for example, nutrient rich herb‐rich forest or nutrient poor barren heath forest, across the country (Appendix S1: Table S3). Forest habitat types are defined based on the composition of understory vegetation but also the dominance of tree species varies between the different types (Pohjanmies et al., 2020). Soil type (Figure 1i) is a measure of site fertility determining the growing conditions for plants. Mineral soil types are defined based on grain size and majority of the mineral study sites are till. All forest related variables were based on field data that have been collected during the understory vegetation survey in 2021–2023 following the guidelines of the NFI.

### Statistical analyses

We used joint species distribution models to investigate species–species and species–environment relationships utilizing R‐package Hmsc (Tikhonov et al., 2020). We modeled the species occurrences at the quadrat level using probit Hmsc models jointly for all species. The models were fitted separately for mineral and peatland sites and for each bioclimatic zone (hemiboreal + south boreal, middle boreal and north boreal) to consider variation in the understory communities across Finland. We used two modeling approaches for identifying the potential indicator species.

In the first approach (null model), we included in the model only species‐specific intercepts and spatially independent site random effects jointly for all the species using the latent factor structure in Hmsc. To identify species that were most strongly linked to the occurrence of the other species, we calculated for each species how much knowing its site random effects reduced the total variance of the site random effects of the other species (see Text box S1 in Appendix S1). To build a list of candidate indicator species, we first chose the species that explained most of the variance of the other species and removed its row and column from the covariance matrix. Then we iteratively continued the same process until we got an ordering of the species with decreasing strength of links to the other species.

In the second approach (covariate model), we extended the first model with the climate and forestry variables: GDD59, precipitation, snow cover length, basal area (only in the models for mineral sites), ditching, and time since clear cut as covariates. In addition, we included the habitat type and soil type as random effects. From the fitted models, we calculated the proportion of total variance explained by the environmental covariates for each species. Variance partitioning was then used to order the species according to how much in total the environment explained of the variation in occurrence.

In all analyses, we fitted the model using eight independent chains with 250,000 iterations in each chain, with the number of latent factors adapted during the first 100,000 iterations. We discarded the first 125,000 iterations of each chain as transient and thinned the remaining 125,000 iterations by a factor of 1000 to get a total of 1000 posterior samples from the eight chains. We evaluated the convergence and mixing of the algorithms by calculating the potential scale reduction factor (PSRF) for the model parameters (Brooks & Gelman, 1998; Gelman & Rubin, 1992). If the convergence of the algorithm was not good, we ran longer runs increasing the number of iterations to 1,250,000, the length of the latent factor adaptation to 500,000, transient phase to 625,000, and thinning interval to 5000. The Tjur *R* ^2^ values that measured the explanatory power of the models varied between 0.03 and 0.77, with only minor differences between the models with and without covariates included. The explanatory power was slightly higher on average in peatland sites than in mineral sites, and slightly lower in the south boreal zone than in the other zones (Appendix S1: Figure S1).

To test how well the indicator species reflect the status of the whole plant community, we used the Hmsc models to predict species occurrences under scenarios described below using the same two modeling approaches as presented above. We divided the sites randomly into four folds and then predicted the occurrences of non‐indicator species in each fold in turn. In each case, the sites from the three other folds (75% of the sites) were training sites, and the sites in the focal fold (25% of the sites) were used as testing sites. In the training sites, the observations of both indicator and non‐indicator species were used to train the model in each case. The three prediction scenarios differed in which observations from the test sites were used in addition to the observations from training sites to train the model. In the *full* scenario, no other observations were used in addition to training sites to test how well the model could extrapolate to new sites and to set a lower bound for the quality of the predictions. In the *conditional* scenario, we included the observations of the indicator species from the test sites to the training data, which indicated how well the status of indicator species reflected the whole community. In the *explanatory* scenario, the observations of all species from the test sites were included in the training data to test how well the model fitted to the data and to set an upper bound for the quality of the predictions. The predictive performance of the models was measured with area under the receiver operative characteristic curve (AUC) and Tjur *R* ^2^ values. The comparison of the scenarios was performed separately for mineral and peatland sites and for each boreal zone. Finally, to test how well the indicator species reflect temporal patterns in plant communities, we replicated the predictive performance test using species data from the first understory survey in 1985. We fitted otherwise the same models under all scenarios as with the most recent understory data but removed the time since last clear‐cut variable from the covariate model, because the information was not available for the 1985 data.

## RESULTS

**FIGURE 2:**
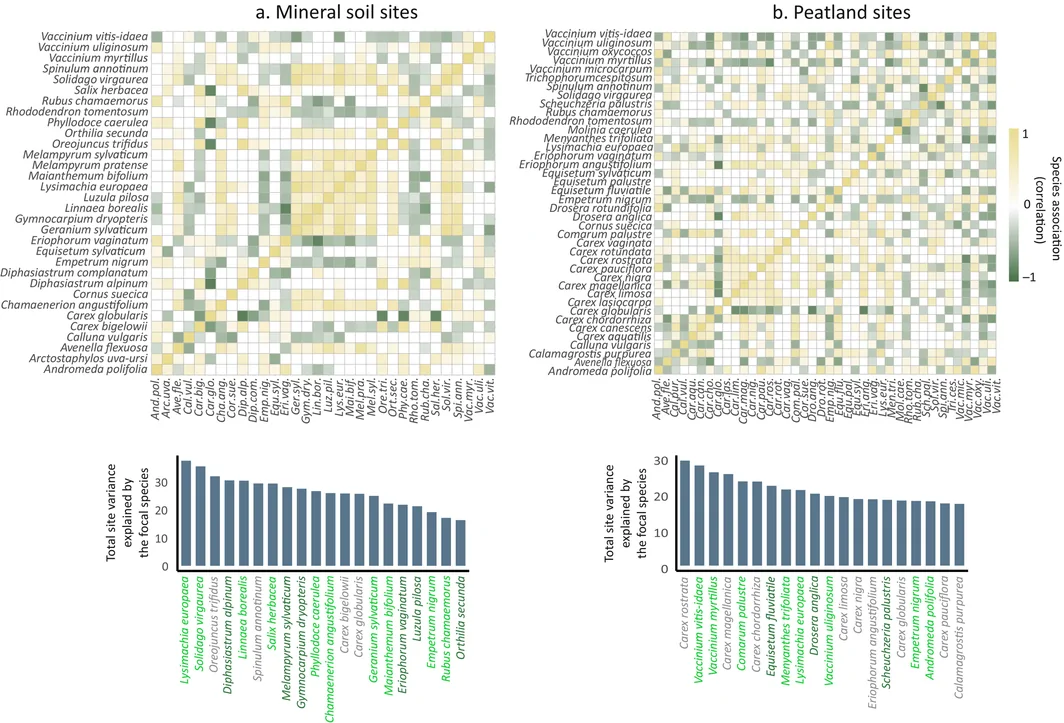
Species associations on mineral soil (a) and on peatland sites (b) in the north boreal zone. Correlation matrices visualize positive (yellow) and negative (green) species‐species associations with color darkness indicating the strength of the association. Bar plots show 20 species with strongest associations (both positive and negative ones included) with the other species based on the explained variance (total site variance among species explained by the focal species ranging 16.3–37.5 for mineral and 17.5–29.7 for peatland sites). In the bar plots, text color represents species identifiability: bright green = easy, dark green = moderate, and gray = difficult. Results for the other zones are presented in Appendix S1.

Our analyses revealed both positive and negative species associations with varying strengths across species that were broadly similar across the bioclimatic zones (Figure 2, Appendix S1: Figures S2 and S3). On mineral sites, negative associations were mainly detected between shrubs (e.g., *Vaccinium* sp.) and herbaceous species (e.g., *Melampyrum* sp.), while positive associations were mostly found within these groups. On peatland sites, the observed patterns were more variable among the plant groups. In the two site types, both shrub and herbaceous species showed strong associations with other species across zones. Some of the highly covarying species were shared between the zones (e.g., *Geranium sylvaticum* on mineral sites) and even between the mineral and peatland sites (e.g., *Empetrum nigrum*) (see bar plots in Figure 2, Appendix S1: Figures S2 and S3). Overall, species differed considerably in their associations with other species (mean absolute correlation ranging between 0.28–0.41 and 0.29–0.43 across zones for mineral and peatland sites, respectively).

**FIGURE 3:**
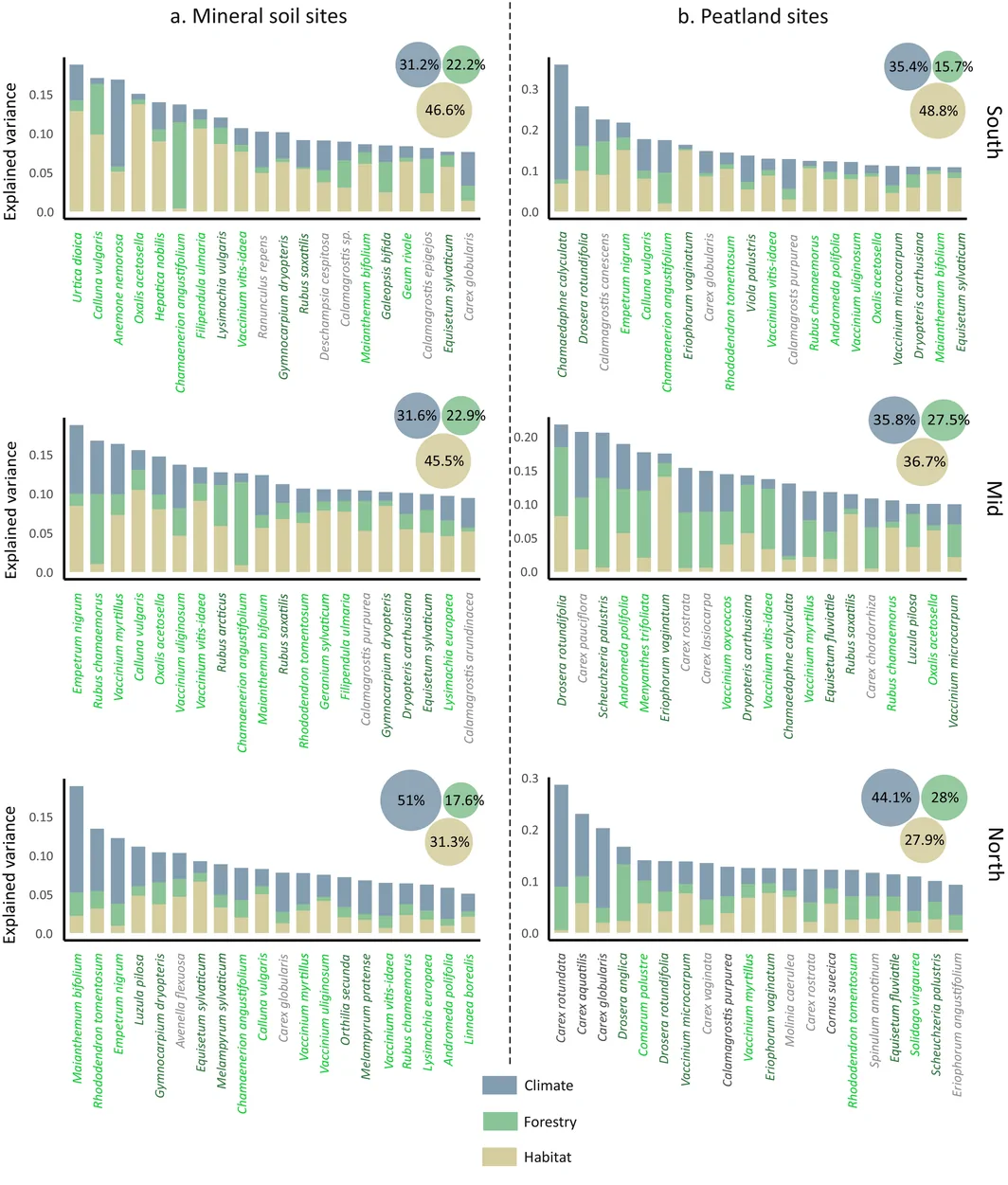
Proportion of variance explained by climate, forestry and habitat variables on mineral soil sites (a) and on peatland sites (b) on different bioclimatic zones for the 20 species with the strongest environmental response. Mean explained variance across the zones was 0.11 and 0.15 for mineral and peatland sites, respectively. The circles show the relative proportion of variance explained by the variable groups across all species in each zone. Species names are colored to represent identifiability: bright green = easy, dark green = moderate and gray = difficult. Climate variables = GDD5, precipitation, and snow, Forest variables = basal area (only in mineral sites), ditching, and time since clear cut, Habitat variables = random effects of habitat and soil type.

The climate and forestry variables and random factors (excluding site to focus on the ecological effects) explained the observed variance more in the models for peatlands than for mineral sites with mean proportion of variance explained of 0.1 and 0.08 across zones, respectively. On mineral sites, most of the variation was explained by the habitat variables (mean relative importance of 41.2%), while the influence of climate variables increased with latitude (31.2%, 31.6%, and 51% for south, middle, and north boreal zones, respectively; Figure 3a). Climate and habitat variables explained comparable amounts of variation on peatland sites (mean 38.5% and 38.0%, respectively), with habitat being relatively more important again in the south (Figure 3b). Species responses to forestry variables were broadly consistent across zones and site types, with a mean relative importance of approximately 20%. From the individual climate and forestry variables species responded most strongly to GDD5, snow, basal area, and ditching on both mineral and peatland sites (Appendix S1: Figure S4). Species responses to environmental conditions varied across the zones, indicating strong context dependency. The proportion of variation explained by the environmental variables differed for the same species depending on the zone (e.g., for *Maianthemum bifolium* on mineral sites and for *Andromeda polifolia* on peatlands). On both site types, species with the strongest environmental response included both shrub and herbaceous species. Some of the species were zone‐specific, such as *Anemone nemorosa* (southern mineral sites) or *Cornus suecica* (northern peatlands), whereas some were shared between the zones and the two site types, for example *V. vitis‐idaea* (Figure 3).

**FIGURE 4:**
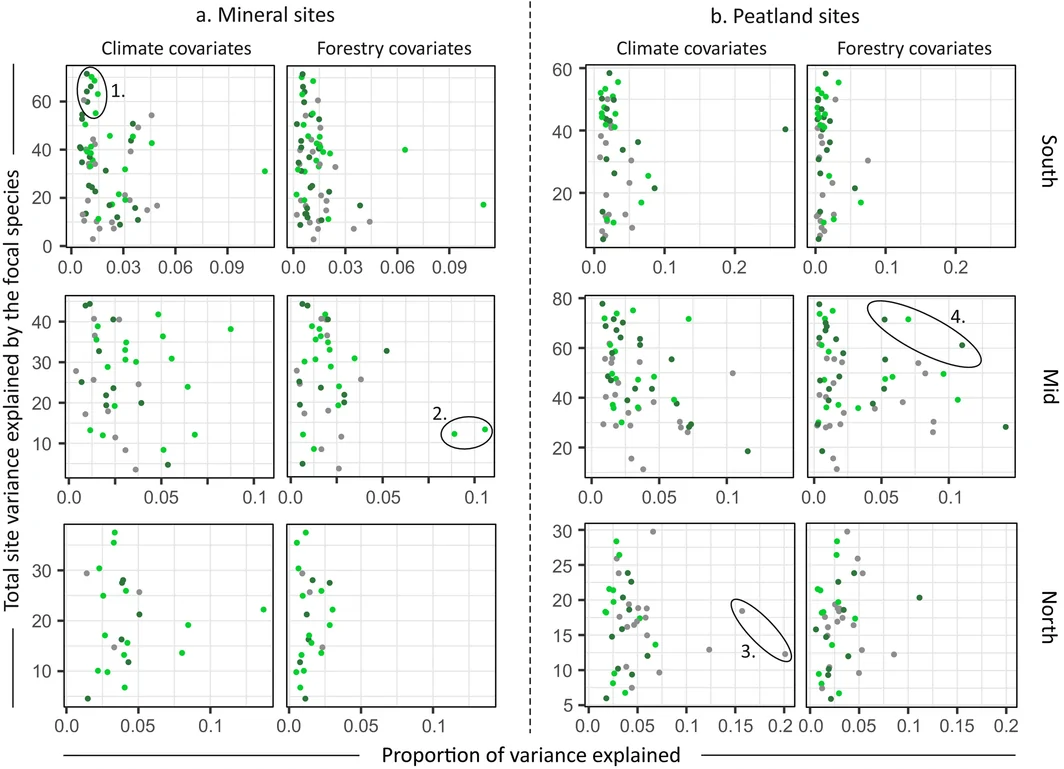
The relationship between species' associations and their response to climate and forestry covariates in mineral (a) and peatland sites (b) in different bioclimatic zones. Dots represent different species, and circled dots represent example species with either strong association with other species (1), strong environmental response (2 and 3) or both (4). Dot color represents species identifiability: Bright green = easy, dark green = moderate and gray = difficult. Climate covariates include the combined effect of GDD5, precipitation, and snow. Forestry covariates include the combined effect of basal area (only in mineral sites), ditching, and time since clear cut. Circled species in (1) are, for example, *Veronica chamaedrys*, *Fragaria vesca* and *Geranium sylvaticum*; *in* (2) *Rubus chamaemorus* and *Chamaenerion angustifolium*; in (3) *Carex globularis* and *Carex rotundata*; and *in* (4) *Luzula pilosa*, *Andromeda polifolia* and *Drosera rotundifolia*.

The effect of climate and forestry variables was also investigated in relation to species associations. This allowed us to further identify species whose occurrence is strongly associated with the occurrence of other species, or primarily driven by climatic conditions or forest structure and, importantly, to characterize species that respond to both environmental and biotic factors (Figure 4).

Based on the results presented above, we compiled a list of potential indicator species across the boreal zones for mineral sites and peatlands that could be useful in forest understory monitoring (Table 1). The lists are based on the zone‐specific ranking of 20 species with the strongest environmental response and 20 species with the strongest association with other species (bar plots in Figures 2 and 3 and Appendix S1: Figures S2 and S3). Species that showed both strong environmental response and association with other species were prioritized, as well as those ranking high in several zones in either of the responses. To support nationwide monitoring and to test the applicability of our results, we developed our model‐derived indicator species list further in cooperation with species experts from the Natural Resources Institute Finland (Luke). The final species list (Appendix S1: Table S4) comprising 25 species based on the analysis results was compiled, combining species from the lists for mineral and peatland sites with a focus on easy identifiability (Table 1). In addition, 10 species were added based on expert opinion considering species conservation status, for example. The resulting list of 35 indicator species was tested in a field survey in summer 2025 as part of a project aiming to find new variables to be measured in the NFI to improve assessment of biodiversity and forestry impacts (VMI‐moni, Luke, 2025).

**TABLE 1:** Potential indicator species across boreal zones on mineral and peatland sites.

Site and species	Environmental response
Mineral sites	
***Andromeda polifolia*** ^1^*	Precip
***Angelica sylvestris*** ^2^	Habitat
*Avenella flexuosa* ^3^*	Habitat
***Calluna vulgaris*** ^1^	Habitat
***Carex globularis*** ^3^*	GDD5, precip
***Chamaenerion angustifolium*** ^1^*	Basal area, precip
***Dryopteris carthusiana*** ^2^*	Habitat
***Empetrum nigrum*** ^1^*	Snow, habitat
***Equisetum sylvaticum*** ^2^	Habitat
***Filipendula ulmaria*** ^1^*	Habitat
***Geranium sylvaticum*** ^1^*	Habitat
*Geum rivale* ^1^*	Habitat
***Gymnocarpium dryopteris*** ^2^*	Soil type, habitat
*Hepatica nobilis* ^1^*	Habitat
*Linnaea borealis* ^1^*	Habitat
*Luzula pilosa* ^2^*	Habitat
***Lysimachia europaea*** ^1^*	Habitat, precip
***Maianthemum bifolium*** ^1^*	Habitat, snow
***Melampyrum sylvaticum*** ^2^*	Snow, habitat
***Melica nutans*** ^3^	Habitat
***Orthilia secunda*** ^2^*	GDD5
***Oxalis acetosella*** ^1^*	Habitat
*Ranunculus repens* ^3^*	Snow
***Rhododendron tomentosum*** ^1^*	Precip, habitat
*Rubus arcticus* ^2^*	Habitat
***Rubus chamaemorus*** ^**1**^	Cut class, habitat
***Rubus saxatilis*** ^2^*	Habitat
***Solidago virgaurea*** ^1^	Precip, GDD5
***Spinulum annotinum*** ^2^	Soil type, habitat
***Vaccinium myrtillus*** ^1^	GDD5, habitat
***Vaccinium uliginosum*** ^1^	GDD5, habitat
***Vaccinium vitis‐idaea*** ^1^*	Habitat
Peatland sites	
***Andromeda polifolia*** ^1^*	Ditching, habitat
***Calamagrostis purpurea*** ^3^	Snow, precip
***Carex chordorrhiza*** ^3^*	Ditching
***Carex globularis*** ^3^	GDD5, habitat
***Carex pauciflora*** ^3^*	Snow
***Carex rostrata*** ^3^*	Ditching, GDD5
***Chamaedaphne calyculata*** ^2^	Snow
***Comarum palustre*** ^1^*	Habitat
*Cornus suecica* ^1^*	Soil type
*Drosera anglica* ^2^*	Ditching
***Drosera rotundifolia*** ^2^*	Ditching, habitat
***Dryopteris carthusiana*** ^2^*	Ditching, habitat
***Empetrum nigrum*** ^1^*	Habitat
***Equisetum fluviatile*** ^2^	Snow, habitat
***Equisetum sylvaticum*** ^2^*	Habitat
***Eriophorum vaginatum*** ^2^	Habitat
***Gymnocarpium dryopteris*** ^2^	Habitat
***Luzula pilosa*** ^2^*	Ditching
***Lysimachia europaea*** ^1^	Habitat
***Maianthemum bifolium*** ^1^*	Habitat
*Menyanthes trifoliata* ^1^*	Ditching
***Oxalis acetosella*** ^1^	Habitat
***Rhododendron tomentosum*** ^1^*	Cut, habitat
*Rubus chamaemorus* ^1^*	Habitat
***Rubus saxatilis*** ^2^*	Habitat
***Scheuzchzeria palustris*** ^2^*	Ditching
***Solidago virgaurea*** ^1^	Snow, GDD5
***Vaccinium microcarpum*** ^2^	Ditching, habitat
***Vaccinium myrtillus*** ^1^*	Habitat
***Vaccinium oxycoccos*** ^2^*	GDD5
***Vaccinium vitis‐idaea*** ^1^*	Ditching, habitat
*Viola palustris* ^2^*	Snow

*Note*: Species marked with asterisk (*) had both strong environmental response and association with other species at least in one boreal zone. Bolded species showed either strong environmental response or association with other species in several zones. Superscript in species names represents identifiability: ^1^ = easy, ^2^ = moderate, ^3^ = difficult. Environmental response shows the most influential environmental variable(s) for species across the zones in which it was ranked to top 20.

The compiled list (excluding the few species that were added by Luke but were not present in the modeling data) was also used to test how well the chosen indicator species reflect the status of the whole understory community. With the covariate model the AUC values for the *conditional* scenario (average 0.81) were between the average values for the *explanatory* (0.97) and *full* scenarios (0.73), showing that the chosen indicator species can predict the occurrence of non‐indicator species across the boreal zones (Appendix S1: Figure S5). The average AUC for the *conditional* scenario under the null model (0.79) was higher than the average for the *full* scenario under covariate model (0.73), which indicates that knowing the status of the indicator species leads to better understanding of the whole community than the environmental conditions of the site. Similar patterns were also seen in the Tjur R^2^ values, which were highest for the *explanatory* scenario (averages covariate: 0.34, null: 0.35), lowest for the *full* scenario (covariate: 0.04, null: 0) and in‐between for the *conditional* scenario (covariate: 0.11, null: 0.96). The prediction performance test showed similar results when the models were fitted with the 1985 data suggesting that the chosen indicator species could reflect patterns in the understory communities with decadal time span.

## DISCUSSION

Effective biodiversity monitoring requires indicators that reflect both species–environment relationships and biotic context, yet these aspects are rarely integrated across broad ecological gradients. Our study addresses this gap by combining species covariance and environmental response modeling across forest types, management histories, and bioclimatic zones. This dual approach allows us to identify species that are informative of both local community composition and broader environmental patterns, offering a robust and scalable tool for selecting indicator species.

In the investigated understory plant species pool, the species with high positive correlation with other species were mostly herbaceous and associated with other herbaceous species, both on mineral and peatland sites and across bioclimatic zones. Respectively, dwarf shrubs showed positive associations with each other but negative with many herbaceous species indicating variation in species' niche preferences and hence the effect of forest habitat type which represents variation in site fertility. These results corroborate with findings that niche optima for herbaceous and shrub species are at the different ends of the fertility gradient (Heikkinen & Mäkipää, 2010). Furthermore, strong species associations can indicate the presence of facilitator or competitor species as species' realized niches are restricted by species biotic interactions (Wisz et al., 2013). For example, crowberry (*E. nigrum*) is a strong resource competitor with allelopathic effects restricting the species pool capable to co‐exist with it (Tybirk et al., 2000). Regarding the approach used to capture species associations, ranking species based on their covariance with other species represents co‐occurrence patterns rather than actual biotic interactions (Blanchet et al., 2020). However, this can be particularly useful when looking for species representing a wider community, as in a long‐term monitoring scheme considered here. Selecting both positively and negatively associated species as indicators could therefore ensure covering different environmental niches and community compositions. However, we acknowledge that to detect species associations more thoroughly inspecting temporal changes in the associations would be beneficial.

Looking at the abiotic effects the importance of forest habitat type was evident likely because it can represent several ecological aspects, such as nutrient and moisture conditions, and to some extent also forest structure (Heikkinen & Mäkipää, 2010; Hotanen et al., 2008), which possibly decreased the effect of individual climate and forestry factors. However, the other environmental variables were also influential. Overall, climate variables explained more variance than forestry variables, with snow cover duration often being the most important climatic predictor. The strong effect of snow likely relates to its ability to reflect large‐scale latitudinal and longitudinal climate patterns in interplay with local topography and forest structure due to its uneven distribution in a landscape (Williams et al., 2015). The effect of snow was observed both on mineral and peatland sites aligning with previous studies analyzing plant distributions in the north (Antão et al., 2022; Rissanen et al., 2021). Species with strong climatic, and especially snow related, responses (e.g., *E. nigrum* as suggested by our results) could be important indicator species to monitor in high latitudes where winter conditions are expected to change more drastically than summer conditions (Rantanen et al., 2022).

From the forestry variables, basal area and ditching were the most important ones in mineral and peatland sites, respectively, whereas time since clear cut had small influence despite its major disturbance effect on forest ecosystem (Kaarlejärvi et al., 2021). The finding is less surprising on peatlands where ditching is the greatest management related disturbance affecting the entire ecosystem due to changes in water table and soil properties (Sarkkola et al., 2010). Furthermore, in Finland, the majority of peatland forests (~70% of our peatland study sites) are drained (Korhonen et al., 2024) to enhance forest growth. On mineral soil sites, basal area likely captures forest management history at least partly as it tends to follow forest age, that is, basal area is higher in older and uncut forests than in those more recently cut (Tonteri et al., 2016). Furthermore, variation in basal area can also represent forest succession. This can be observed in the constant response of fireweed (*Chamaenerion angustifolium*) as the species occurs in open forests during the early successional phase. Moreover, considering the effect of forest conditions together with the climate variables is important when selecting potential indicator species to detect biodiversity change, as they represent interdependent effects on biodiversity (Montràs‐Janer et al., 2024), and species are often more related to one than another like our results suggest.

Our results show that in many cases, species were more strongly linked either to environment or co‐occurrence, calling for the complementary use of the approaches. Different species were also identified based on the approach, suggesting that considering only species co‐occurrences or environmental responses in identifying potential indicator species would provide inadequate information on the community dynamics. Respectively, monitoring species ranking high with both aspects would be particularly interesting due to their potential to reflect both biotic and abiotic conditions and possible changes in them. Furthermore, species ranking high in their responses across bioclimatic zones could be especially useful when considering nationwide monitoring. However, the environmental conditions and species occurrences differ across latitudinal gradients (Antão et al., 2022), causing changes to species' environmental responses and the strength of species associations between different zones, as our results showed. Species' ability to indicate the status of a whole community can hence vary across the country (Skogsstyrelsen, 2023), which makes also zone‐specific indicator species useful.

Another important criterion for selecting indicator species is their identifiability in the field, which determines their practical utility in ecological monitoring. For example, in our analysis, sedges (*Carex* spp.) frequently emerged as top candidates—especially in the peatland habitats—due to their strong species associations and environmental responsiveness. However, the accurate identification of these species is challenging and time consuming. In monitoring programs constrained by limited resources and frequent staff turnover, indicator species should be easily recognizable and robust to varying levels of taxonomic expertise (Sato et al., 2019). Furthermore, combining the results from different models to select potential indicator species, like we did here, inevitably involves subjective choices, such as prioritizing the importance of field identifiability or species geographical occurrence. Therefore, the final selection of indicator species is always context‐dependent and guided by the monitoring target in relation to which the chosen species should also be assessed and accordingly revised (Bal et al., 2018).

In the NFI related field survey in summer 2025, the applicability of the proposed indicator species list was tested and 28 out of 35 species were observed in the sample plots. Species not observed in the plots included mostly the endangered and invasive species added to the list based on expert opinion. However, the test sampling of 143 plots covered only a fraction of the 9400 plots sampled annually in the NFI. Incorporating the suggested 35 indicator species added approximately 20 min of work to the tree and site measurements of NFI per plot. This would increase the average cost of current 255 euros (covering field work expenses from forest measurements, traveling, and accommodation) by an estimated 44 euros per plot, though the additional cost would likely be higher, as longer measurement time per plot would increase the total number of fieldwork days and total expenses of the inventory. For comparison, the nationwide vegetation inventory conducted in 2021–2023—including comprehensive assessments of plant community and tree measurements across 2973 sample plots—had an estimated cost exceeding 600 € per plot. This is not to say that the nationwide vegetation inventories of the whole understory community should be replaced with surveys of indicator species, but if added to the current NFI scheme they could offer a complementary approach to increase the temporal frequency and spatial coverage of the understory data in a cost‐efficient manner. Moreover, the presented species list is not exhaustive and could be modified to better meet the available inventory resources by revising the number of species, for example.

In conclusion, our study offers a comprehensive and adaptable approach for selecting indicator species by jointly considering species co‐occurrence and environmental responses across diverse ecological settings. This integrated perspective enables the identification of species that reflect both local community structure and broader environmental change—key for meaningful long‐term monitoring. Even though our analyses did not include temporal change in species associations or environmental response per se, the results of the predictive performance test indicated robustness in the selection of the potential indicator species regardless of the used data. The selected indicators predicted species community both in 1985 and in current conditions suggesting capability to reflect community patterns over several decades, promoting their suitability to long‐term monitoring in the future. While our analysis focused on vascular plants, the approach is readily transferable to other taxa. By combining ecological relevance with feasibility, this framework supports more targeted and informed biodiversity monitoring, contributing to improved understanding and stewardship of forest ecosystems under accelerating climate and intensifying management regimes.

## AUTHOR CONTRIBUTIONS

Tuuli Rissanen wrote the first draft of the manuscript and Jukka Sirén conducted the analyses. Jarno Vanhatalo and Anna‐Liisa Laine conceptualized the study with the contribution of Raisa Mäkipää, Tuuli Rissanen, and Jukka Sirén All authors had equal major contribution to the initial research design and formulation of the manuscript. All authors contributed to the writing of the manuscript.

## CONFLICT OF INTEREST STATEMENT

The authors declare no conflicts of interest.

## Supporting information


Appendix S1:

## Data Availability

Data and code (Rissanen, 2026) are available in Zenodo at https://doi.org/10.5281/zenodo.19663305.
