# Supplementary material for: Informing indicator species selection by combining species associations and environmental responses
Source: Ecol Appl. 2026 Aug 20;36(5):e70296. doi: 10.1002/eap.70296 (PMC13491413; doi:10.1002/eap.70296)
Supplement: Supplementary file 1 [file EAP-36-e70296-s001.pdf]

Appendix S1

Informing indicator species selection by combining species associations and environmental responses

Tuuli Rissanen, Jukka Sirén, Raisa Mäkipää, Jarno Vanhatalo & Anna-Liisa Laine

Ecological Applications

Table S1. *Species used in the analyses of mineral sites with their zone-specific and total prevalence. Identifiability is an expert-knowledge-based evaluation on species-level identifiability in the field (1 = easy, 2= moderate, 3= difficult). Species names follow the nomenclature of accepted scientific names for vascular plants of The Plant List (http://www.theplantlist.org/).*

Species	Prev. S	Prev. M	Prev. N	Total	Identifiability
Aegopodium podagraria	39	0	0	39	2
Agrostis capillaris	188	90	0	278	3
Andromeda polifolia	0	25	49	74	1
Anemone nemorosa	89	0	0	89	1
Angelica sylvestris	92	28	0	120	2
Anthriscus sylvestris	53	0	0	53	3
Arctostaphylos uva-ursi	0	0	22	22	1
Athyrium filix-femina	129	0	0	129	2
Avenella flexuosa	1803	1292	580	3675	3
Calamagrostis arundinacea	1194	171	0	1365	3
Calamagrostis canescens	36	0	0	36	3
Calamagrostis epigejos	111	50	0	161	3
Calamagrostis purpurea	146	137	0	283	3
Calamagrostis sp	30	0	0	30	3
Calluna vulgaris	495	584	253	1332	1
Carex bigelowii	0	0	47	47	3
Carex canescens	0	38	0	38	3
Carex digitata	114	0	0	114	3
Carex globularis	200	439	62	701	3
Carex sp.	30	0	0	30	3
Chamaenerion angustifolium	384	353	63	800	1
Cirsium helenioides	30	0	0	30	2
Convallaria majalis	311	0	0	311	1
Cornus suecica	0	30	31	61	1
Deschampsia cespitosa	176	86	0	262	3
Diphasiastrum alpinum	0	0	17	17	2
Diphasiastrum complanatum	0	0	18	18	2
Dryopteris carthusiana	607	186	0	793	2
Dryopteris expansa	142	0	0	142	2
Empetrum nigrum	80	623	835	1538	1
Equisetum arvense	30	0	0	30	2
Equisetum sylvaticum	484	231	65	780	2
Eriophorum vaginatum	0	27	16	43	2
Festuca ovina	33	0	0	33	3
Filipendula ulmaria	97	35	0	132	1
Fragaria vesca	223	0	0	223	1
Galeopsis bifida	28	0	0	28	2
Geranium sylvaticum	82	46	27	155	1
Geum rivale	40	0	0	40	1
Goodyera repens	26	0	0	26	1
Gymnocarpium dryopteris	381	131	21	533	2

Hepatica nobilis	43	0	0	43	1
Hieracium sp	39	0	0	39	3
Hypericum maculatum	33	0	0	33	2
Juncus filiformis	28	0	0	28	3
Linnaea borealis	361	264	148	773	1
Luzula pilosa	935	642	107	1684	2
Lysimachia europaea	1324	578	79	1981	1
Lysimachia vulgaris	35	0	0	35	2
Maianthemum bifolium	1476	437	20	1933	1
Melampyrum pratense	840	560	84	1484	2
Melampyrum sylvaticum	382	163	23	568	2
Melica nutans	90	21	0	111	3
Milium effusum	67	0	0	67	3
Oreojuncus trifidus	0	0	27	27	3
Orthilia secunda	78	70	18	166	2
Oxalis acetosella	784	107	0	891	1
Paris quadrifolia	27	0	0	27	1
Phegopteris connectilis	34	0	0	34	2
Phyllodoce caerulea	0	0	58	58	1
Potentilla erecta	118	0	0	118	2
Pteridium aquilinum	178	0	0	178	2
Ranunculus repens	57	0	0	57	3
Rhododendron tomentosum	32	295	253	580	1
Rubus arcticus	24	46	0	70	2
Rubus chamaemorus	0	39	50	89	1
Rubus saxatilis	470	106	0	576	2
Salix herbacea	0	0	19	19	1
Solidago virgaurea	316	286	116	718	1
Spinulum annotinum	48	49	54	151	2
Taraxacum officinale	26	0	0	26	1
Taraxacum sp.	28	0	0	28	1
Urtica dioica	56	0	0	56	1
Vaccinium myrtillus	2580	2047	865	5492	1
Vaccinium sp.	66	25	0	91	1
Vaccinium uliginosum	44	435	430	909	1
Vaccinium vitis-idaea	2412	2202	972	5586	1
Veronica chamaedrys	86	0	0	86	2
Veronica officinalis	92	0	0	92	2
Viola canina	36	0	0	36	2
Viola epipsila	0	21	0	21	2
Viola mirabilis	28	0	0	28	2
Viola palustris	55	0	0	55	2
Viola riviniana	152	0	0	152	2

Table S2. *Species used in the analyses of peatland sites with their zone-specific and total prevalence. Identifiability is an expert-knowledge-based evaluation on species-level identifiability in the field (1 = easy, 2= moderate, 3= difficult). Species names follow the nomenclature of accepted scientific names for vascular plants of The Plant List (http://www.theplantlist.org/).*

Species	Prev. S	Prev. M	Prev. N	Total	Identifiability
Agrostis capillaris	23	0	0	23	3
Andromeda polifolia	105	740	311	1156	1
Athyrium filix-femina	15	0	0	15	2
Avenella flexuosa	165	230	43	438	3
Calamagrostis arundinacea	72	29	0	101	3
Calamagrostis canescens	17	38	0	55	3
Calamagrostis purpurea	87	125	28	240	3
Calluna vulgaris	109	426	60	595	1
Carex aquatilis	0	0	23	23	3
Carex canescens	21	72	29	122	3
Carex chordorrhiza	0	41	17	58	3
Carex globularis	190	793	141	1124	3
Carex lasiocarpa	0	90	52	142	3
Carex limosa	0	51	47	98	3
Carex magellanica	0	58	67	125	3
Carex nigra	26	55	13	94	3
Carex pauciflora	0	139	73	212	3
Carex rostrata	19	118	94	231	3
Carex rotundata	0	0	14	14	3
Carex sp.	19	0	0	19	3
Carex vaginata	0	0	19	19	3
Chamaedaphne calyculata	95	323	0	418	2
Chamaenerion angustifolium	75	104	0	179	1
Comarum palustre	21	71	68	160	2
Cornus suecica	0	0	19	19	1
Deschampsia cespitosa	38	54	0	92	3
Drosera anglica	0	0	30	30	2
Drosera rotundifolia	32	182	88	302	2
Dryopteris carthusiana	233	258	0	491	2
Dryopteris expansa	32	0	0	32	2
Empetrum nigrum	146	974	345	1465	1
Equisetum fluviatile	0	54	69	123	2
Equisetum palustre	0	40	38	78	2
Equisetum sylvaticum	188	291	72	551	2
Eriophorum angustifolium	0	0	44	44	3
Eriophorum vaginatum	240	958	224	1422	2
Galium palustre	16	0	0	16	2
Gymnocarpium dryopteris	55	68	0	123	2
Juncus filiformis	15	34	0	49	3
Linnaea borealis	43	50	0	93	1
Luzula pilosa	100	134	0	234	2
Lysimachia europaea	232	335	38	605	1
Maianthemum bifolium	169	79	0	248	1
Melampyrum pratense	80	140	0	220	2
Melampyrum sylvaticum	48	45	0	93	2
Menyanthes trifoliata	0	86	85	171	1

Molinia caerulea	0	132	28	160	3
Orthilia secunda	0	34	0	34	2
Oxalis acetosella	105	37	0	142	1
Potentilla erecta	18	0	0	18	2
Rhododendron tomentosum	162	786	265	1213	1
Rubus arcticus	0	50	0	50	2
Rubus chamaemorus	169	783	321	1273	1
Rubus saxatilis	24	24	0	48	2
Scheuchzeria palustris	0	73	17	90	2
Solidago virgaurea	22	29	20	71	1
Spinulum annotinum	30	41	20	91	2
Trichophorum cespitosum	0	136	97	233	3
Vaccinium microcarpum	22	183	93	298	2
Vaccinium myrtillus	527	1133	245	1905	1
Vaccinium oxycoccos	111	575	216	902	2
Vaccinium sp.	0	22	0	22	2
Vaccinium uliginosum	192	1050	373	1615	1
Vaccinium vitis-idaea	565	1476	255	2296	1
Viola epipsila	0	23	0	23	2
Viola palustris	35	41	0	76	2

Table S3. *Forest habitat types in mineral and peatland sites. Habitat type varies from nutrient richest (1) to nutrient poorest (8) both on mineral and peatland sites.*

Habitat class	Habitat type in mineral sites	Finnish name	Habitat type in peatland sites	Finnish name
1	Herb-rich forests	Lehdot	Rich fens	Letot ja lehtomaiset suot
2	Herb-rich heath forests	Lehtomaiset kankaat	Herb-rich mires	Ruohoiset suot
3	Mesic heath forests	Tuoreet kankaat	Tall-sedge fens and *Vaccinium myrtillus* spruce mires	Suursaraiset ja mustikkaiset suot
4	Sub-xeric heath forests	Kuivahkot kankaat	Pine mires and *Vaccinium vitis-idaea* spruce mires	Piensaraiset ja puolukkaiset suot
5	Xeric heath forests	Kuivat kankaat	Dwarf shrub pine bogs	Tupasvillaiset ja isovarpuiset suot
6	Barren heath forests	Karukkokankaat	Sphagnum bogs and fens	Rahkaiset suot
7	Rock outcrops and sand lands	Kalliomaat ja hietikot	Rock outcrops and sand lands	Kalliomaat ja hietikot
8	Fell forests and heaths	Lakimetsät ja tunturit	Fell forests and heaths	Lakimetsät ja tunturit

Text box S1. Calculating species associations based on the reduction in the total variance of the site random effects

We calculated the proportion of site variance explained by a species using the species-by-species covariance matrix $\Omega = (\omega_{ij})$ of the site random effect. Conditional on knowing the site random effect of a species *j,* the site variance of a species *i* becomes

$$\omega_{ii} - \frac{\omega_{ij}\omega_{ji}}{\omega_{jj}},$$

where the second term is the reduction in the marginal variance. Based on the above formula, we define the total site variance explained by species *j* as the sum of reductions in the site variances of all the other species

$$\sum_{i \neq j} \frac{\omega_{ij}\omega_{ji}}{\omega_{jj}}.$$

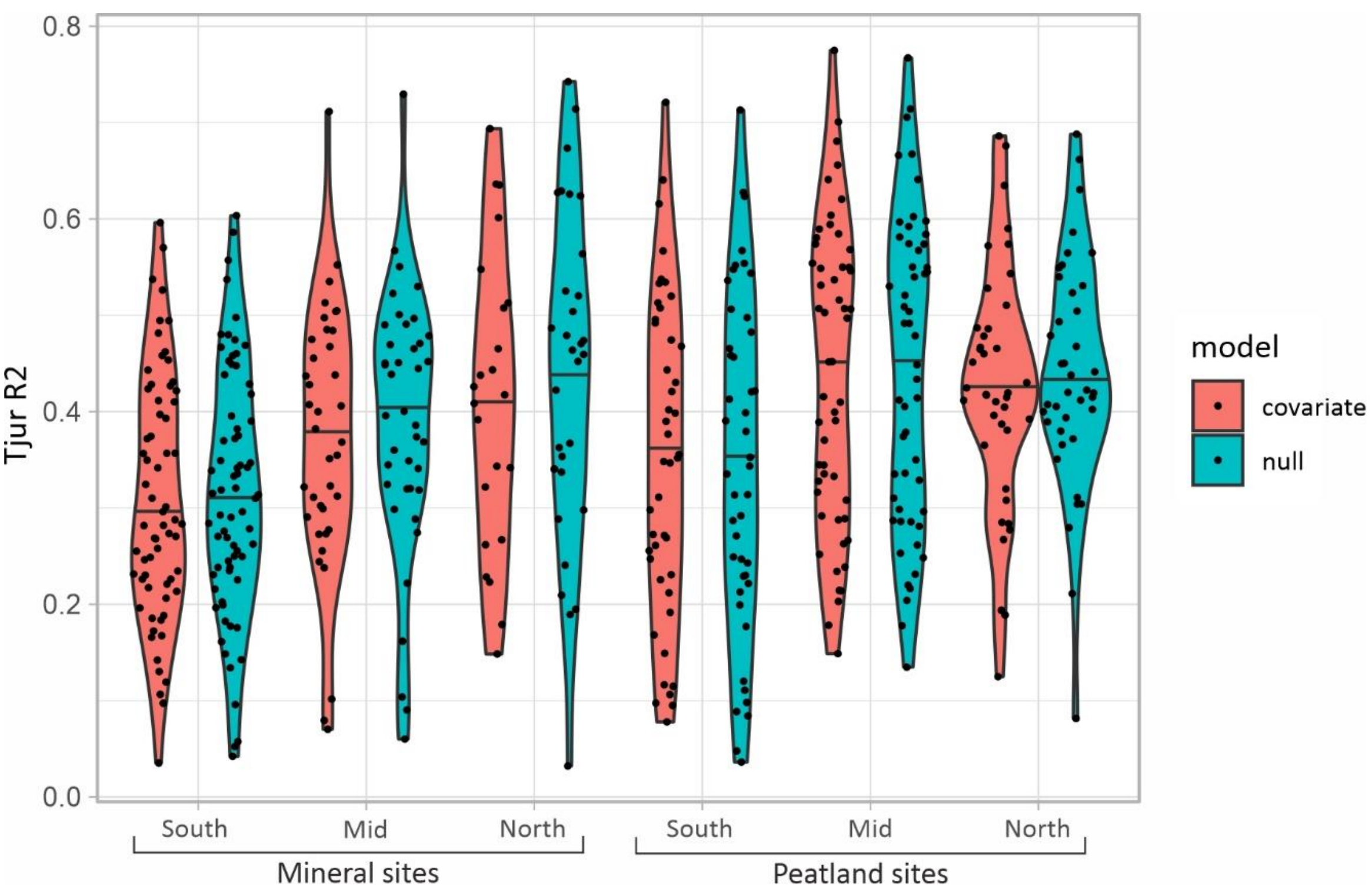

Figure S1. Variation in the model explanatory power (Tjur R2) on mineral and peatland sites in different boreal zones. Models with environmental covariates (climate and forestry variables and habitat random effects) are shown in red and models with only site random effects in blue. Dots represent individual species.

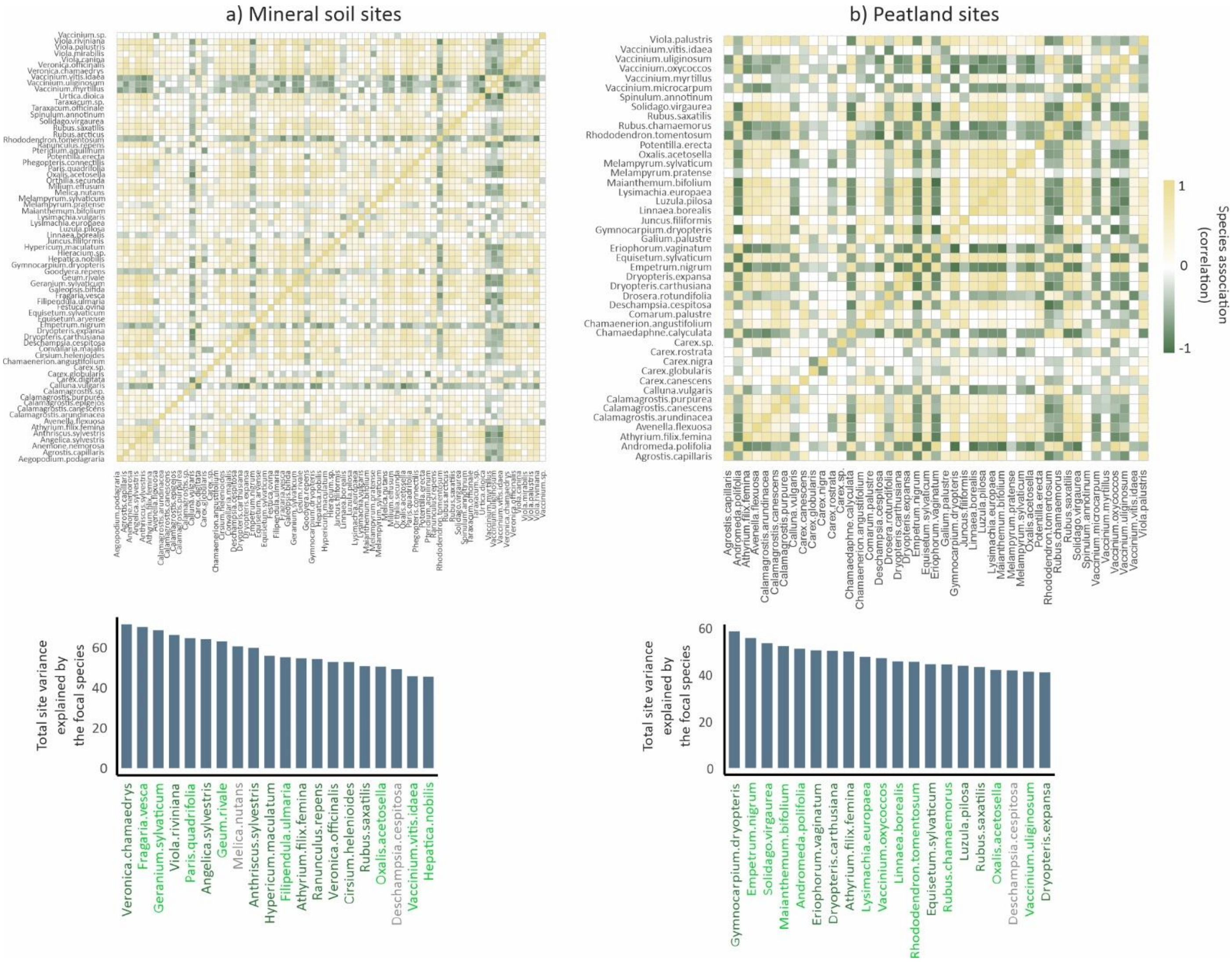

Figure S2. Species associations in south boreal zone on mineral soil (a) and on peatland sites (b). Correlation matrices visualise positive (yellow) and negative (green) species-species associations with colour darkness indicating the strength of the association. Bar plots show 20 species with strongest associations (both positive and negative ones included) with the other species based on the variance reduction (total site variance explained by the focal species ranging 45.5-71.5 for mineral and 40.8-58.4 for peatland sites). In the bar plots text colour represents species identifiability: bright green= easy, dark green= moderate and grey = difficult.

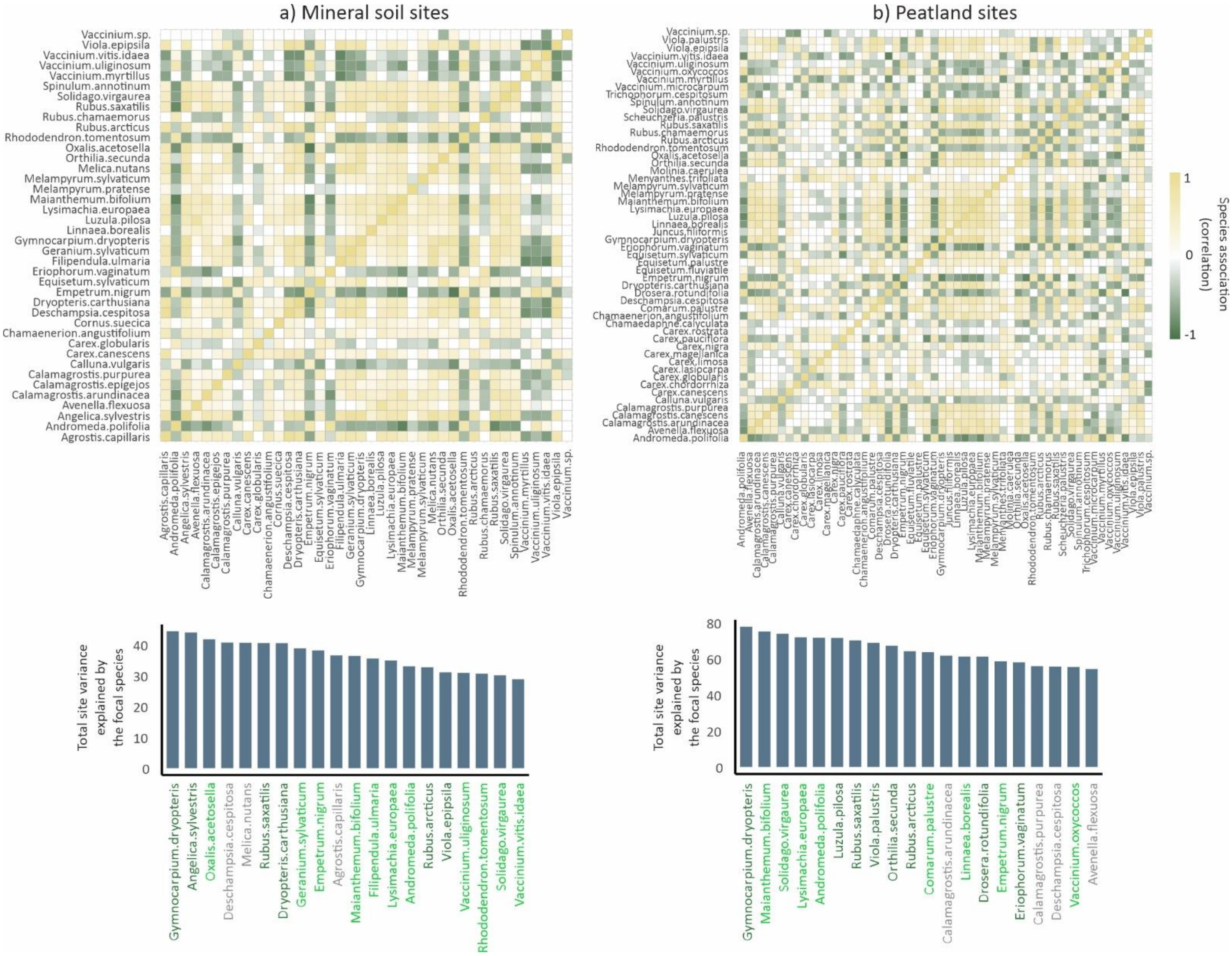

Figure S3. Species associations in middle boreal zone on mineral soil (a) and on peatland sites (b). Correlation matrices visualise positive (yellow) and negative (green) species-species associations with colour darkness indicating the strength of the association. Bar plots show 20 species with strongest associations (both positive and negative ones included) with the other species based on the variance reduction (total site variance explained by the focal species ranging 28.8-44.4 for mineral and 54.3-77.8 for peatland sites). In the bar plots text colour represents species identifiability: bright green= easy, dark green= moderate and grey = difficult.

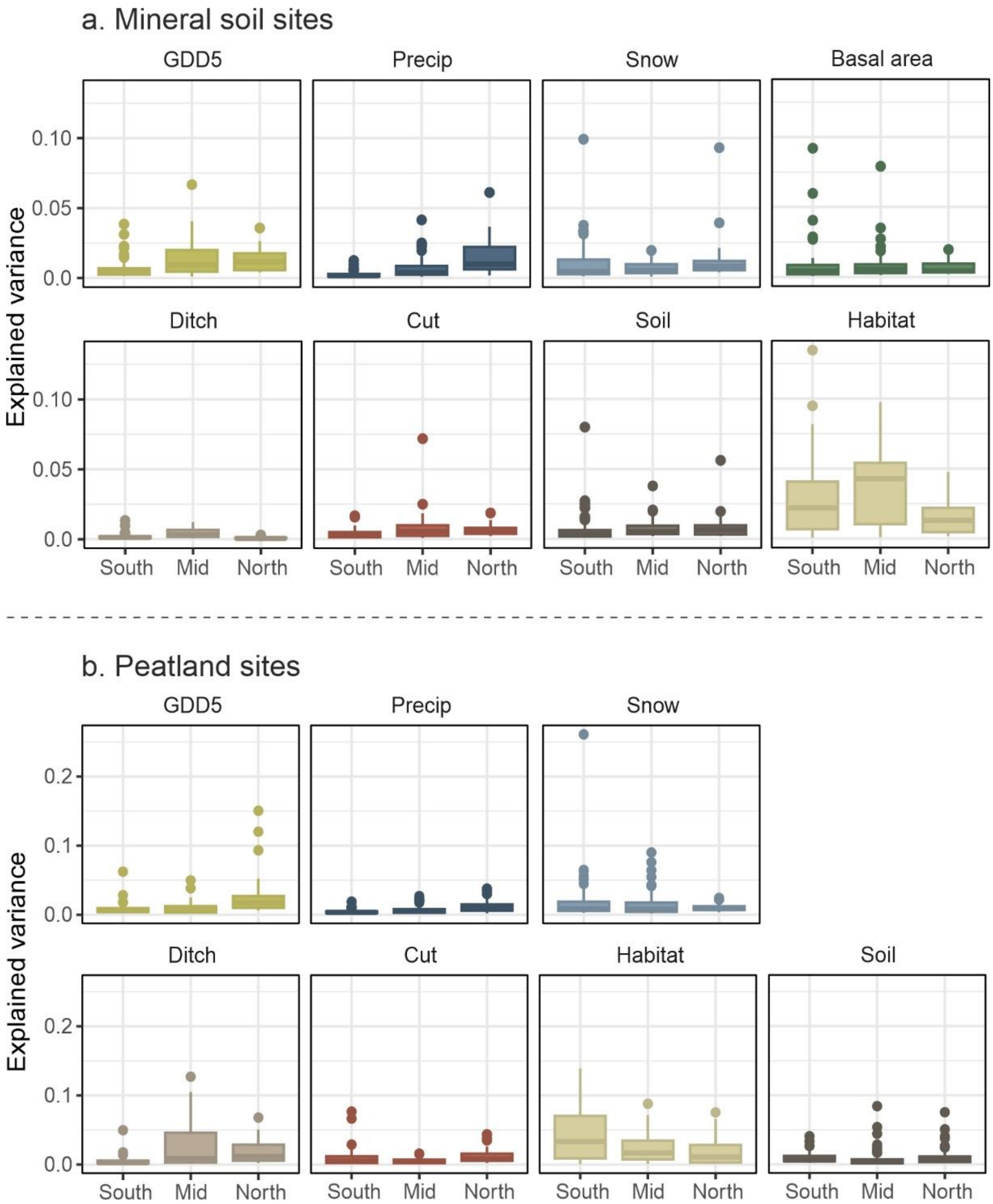

Figure S4. Explained variance by the environmental variables across species on each bioclimatic zones on mineral soil sites (a) and on peatland sites (b). Basal area was not included in the model for peatland sites and is therefore missing from panel b.

Table S4. *Indicator species list compiled by combining the analysis results from mineral and peatland sites across the three boreal zones (Source= JSDM) with expert-based species selection by the Natural Resources Institute Finland (Source= Luke). All species selected based on the analysis results showed a strong environmental response either on the mineral and/or peatland sites. The bolded species had a strong environmental response across several zones at least in one of the site types. Asterisk (*) in species names indicates strong association with other species at least on one zone and site type. Species identifiability: 1= easy, 2= moderate, 3= difficult. Species occurrence information shows the main distribution of a species and is based on the Finnish biodiversity info facility (laji.fi) and marked using the boreal zones: SB= south boreal, MB= middle boreal and NB= north boreal. Species in purple font (Source=Luke) were not used in the model predictive performance test because they were not present in the modelling data.*

Indicator species	Source	Environmental response or other reason for indicator selection	Identifiability	Occurrence
Aconitum lycoctonum	Luke	endangered species	2	MB
Andromeda polifolia*	JSDM	precipitation, ditching, habitat	1	SB, MB, NB
*Avenella flexuosa**	JSDM	habitat	3	SB, MB, NB
Calluna vulgaris	JSDM	habitat	1	SB, MB, NB
Calypso bulbosa	Luke	endangered species	1	MB, NB
Chamaedaphne calyculata	JSDM	snow	2	SB, MB, NB
Chamaenerion angustifolium*	JSDM	basal area, precipitation	1	SB, MB, NB
Comarum palustre*	JSDM	habitat	1	SB, MB, NB
Cornus suecica	JSDM	soiltype	1	SB, MB, NB
Empetrum nigrum*	JSDM	snow, habitat	1	SB, MB, NB
Equisetum sylvaticum	Luke	peatland restoration	2	SB, MB, NB
Fragaria vesca	Luke	settlement and grazing history	1	SB, MB, NB
Gymnocarpium dryopteris*	JSDM	soiltype, habitat	2	SB, MB, NB
*Hepatica nobilis**	JSDM	habitat	1	SB, MB
Hylocomium splendens	Luke	moss species indicating forest management	2	SB, MB, NB
Impatiens glandulifera	Luke	invasive species	1	SB, MB
*Linnaea borealis**	JSDM	habitat	1	SB, MB, NB
Lupinus polyphyllus	Luke	invasive species	1	SB, MB
Luzula pilosa*	JSDM	habitat, ditching	2	SB, MB, NB
Lysimachia europaea*	JSDM	habitat, precipitation	1	SB, MB, NB
Maianthemum bifolium*	JSDM	habitat, snow	1	SB, MB, NB
Melampyrum sylvaticum*	JSDM	snow, habitat	2	SB, MB, NB
*Menyanthes trifoliata**	JSDM	ditching	1	SB, MB, NB
Oxalis acetosella*	JSDM	habitat	1	SB, MB, NB
Pulsatilla patens	Luke	endangered species	2	SB
Pulsatilla vernalis	Luke	endangered species	2	SB
Quercus robur	Luke	umbrella species	1	SB, MB
Rhododendron tomentosum*	JSDM	precipitation, cut class, habitat	1	SB, MB, NB
Rubus chamaemorus*	JSDM	cut class, habitat	1	SB, MB, NB
Rubus idaeus	Luke	forest management	1	SB, MB, NB
Rubus saxatilis*	JSDM	habitat	2	SB, MB, NB
Vaccinium microcarpum	JSDM	ditching, habitat	2	SB, MB, NB
Vaccinium myrtillus*	JSDM	GDD5, habitat	1	SB, MB, NB
Vaccinium oxycoccos*	JSDM	GDD5, habitat	2	SB, MB, NB
Vaccinium uliginosum*	JSDM	GDD5, habitat	1	SB, MB, NB
Vaccinium vitis-idaea*	JSDM	ditching, habitat	1	SB, MB, NB

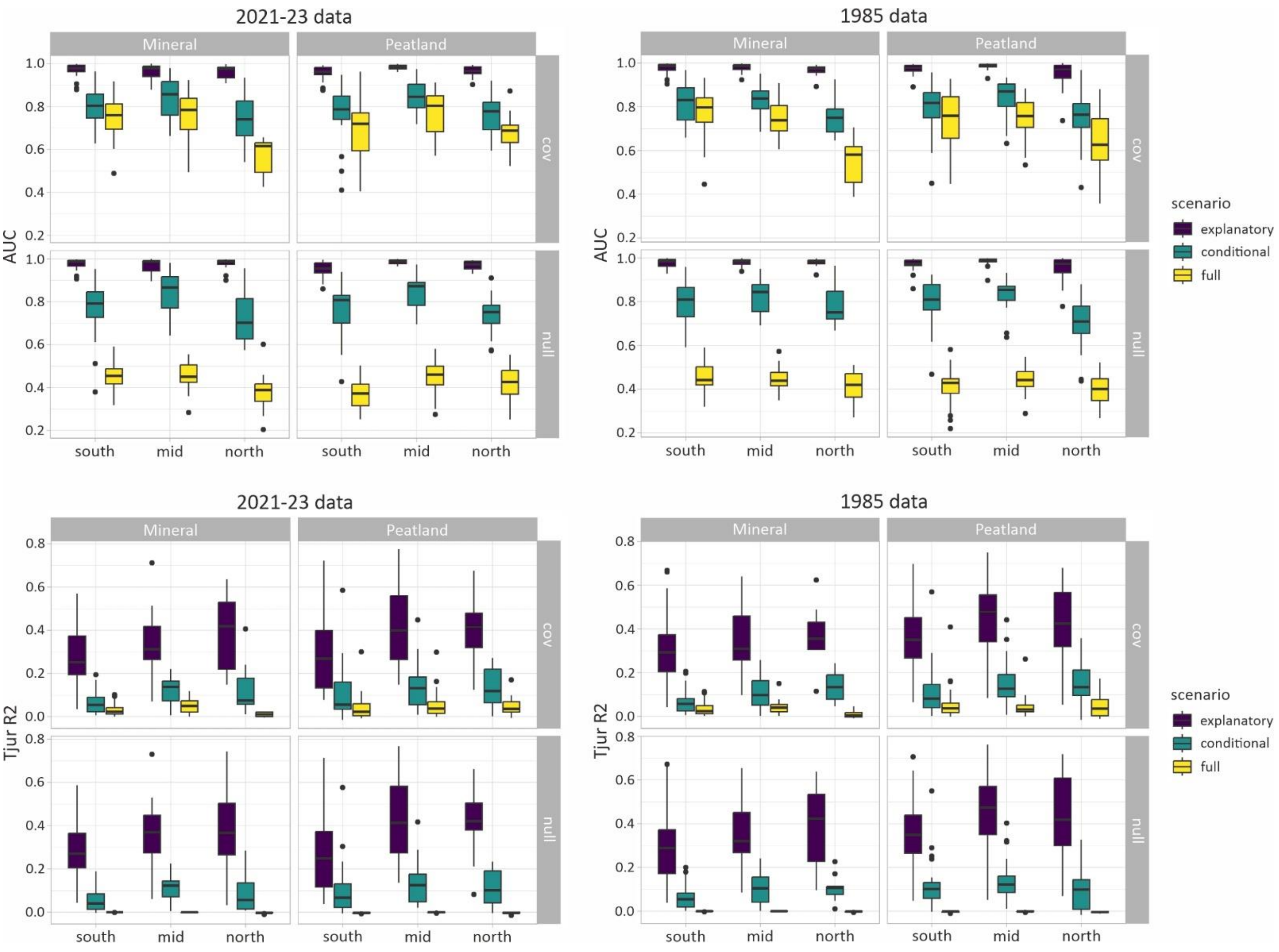

Figure S5. Results of the predictive performance test of the indicator species using the current (2021-23) and past (1985) species data. The Hmsc models were fitted separately for mineral and peatland sites and for each bioclimatic zone (south, mid and north boreal). The models were fitted both with and without environmental covariates (cov and null models, respectively). In each case 75% of the sites were used to train the model and the remaining 25 % of the sites to test the model. The boxplots show variation in the predictive power measured with AUC and Tjur R2 values under the different prediction scenarios. The full scenario in yellow sets the lower bound for the quality of predictions by showing how well the model can extrapolate to new sites, i.e. no other observations were used in addition to training sites. The conditional scenario in turquoise indicates how well the indicator species reflect the whole plant community, i.e. the observations of the indicator species from the test sites were added to the train sites. The explanatory scenario in purple sets the upper bound for the quality of the predictions by showing how well the model fit to the data by using the original models with all possible data.
